# Circular RNA-0007059 protects cell viability and reduces inflammation in a nephritis cell model by inhibiting microRNA-1278/SHP-1/STAT3 signaling

**DOI:** 10.1186/s10020-021-00372-6

**Published:** 2021-09-17

**Authors:** Peng-Wei Guo, Hai-Ting Huang, Jing Ma, Yao Zuo, Dan Huang, Lin-Lin He, Zi-Ming Wan, Cheng Chen, Fa-Fen Yang, Yan-Wu You

**Affiliations:** 1 First Affiliated Hospital of Jinan University, Guangzhou, 510630 Guangdong China; 2 Department of Nephrology, Affiliated Hospital of Youjiang Medical University for Nationalities, No.18 Zhongshan Road II, Baise, 533000 Guangxi Zhuang Autonomous Region China; 3 Department of Nephrology, The First Affiliated Hospital of Chongqing Medical University, Youyi Road 1, Chongqing, 400042 China; 4 Department of Nephrology, Renmin Hospital of Wuhan University, No. 238 Jiefang Road, Wuchang District, Wuhan, 430060 Hubei China

**Keywords:** Nephritis, circ_007059, miR-1278, SHP-1, STAT3

## Abstract

**Background:**

Increasing evidence has indicated that circular RNAs (circRNAs) play a role in various diseases. However, the influence of circRNAs in nephritis remains unknown.

**Methods:**

Microarray analysis and RT-qPCR were used to detect the expression of circRNA. Type I IFN were administrated to RMC and HEK293 cells to establish a nephritis cell model. CCK-8, MTT assay, and flow cytometry were used to assess cell proliferation, viability, and apoptosis of cells. Bioinformatics analysis and dual luciferase reporter assay detect the interaction of circ_0007059, miRNA-1278, and SHP-1. Glomerulonephritis was performed in a mouse model by administration of IFNα-expressing adenovirus. IHC staining showed the pathogenic changes.

**Results:**

In the present study, the expression of circ_0007059 in type I interferon (IFN)-treated renal mesangial cells (RMCs), lupus nephritis (LN) specimens, and HEK293 cells was downregulated compared with that in normal healthy samples and untreated cells. Circ_0007059 overexpression resulted in increased cell proliferation, cell viability, apoptosis, and inflammation-associated factors (CXCL10, IFIT1, ISG15, and MX1) in RMCs and HEK293 cells. In addition, circ_0007059 overexpression significantly restored cell proliferation and viability and inhibited IFN-induced apoptosis. Further, the increased expression resulted in reduced inflammation and the downregulation of CXCL10, IFIT1, ISG15, and MX1 in RMCs and HEK293 cells. Circ_0007059 serves as a sponge for miR-1278 so that the latter can target the 3′-untranslated region of *SHP-1*. Overexpressed circ_0007059 inhibited miR-1278 expression and elevated SHP-1 expression, subsequently reducing STAT3 phosphorylation. Meanwhile, miR-1278 was upregulated and SHP-1 was downregulated in LN samples and IFN-treated cells. The restoration of miR-1278 counteracted the effect of circ_0007059 on viability, apoptosis, and inflammation as well as on SHP-1/STAT3 signaling in RMCs and HEK293 cells. We also investigated the role of SHP-1 overexpression in IFN-treated RMCs and HEK293 cells; SHP-1 overexpression resulted in a similar phenotype as that observed with circ_0007059 expression.

**Conclusions:**

The study indicates that circ_0007059 protects RMCs against apoptosis and inflammation during nephritis by attenuating miR-1278/SHP-1/STAT3 signaling.

**Supplementary Information:**

The online version contains supplementary material available at 10.1186/s10020-021-00372-6.

## Background

Systemic lupus erythematosus (SLE) is a heterogeneous systemic autoimmune disease characterized by extensive clinical features (Tsokos et al. 2016). Lupus nephritis (LN) is one of the worst complications of SLE and a significant indicator of poor prognosis (Zubiria Salgado and Herrera-Diaz 2012; Hahn et al. 2012). Inflammation of the glomeruli and tubules is the most common symptom of LN, which is believed to result from the sediment of immune complexes in the kidney (Davidson and Aranow 2010; Zhen et al. 2005). The subsequent activation of a battery of inflammatory reactions and generation of type I interferon (IFN) are instrumental for studying LN (Rönnblom et al. 2006; Tang et al. 2009; Baechler et al. 2004). Unfortunately, the development of LN in lupus-prone mice requires a long time, and the condition is unstable. This is an impediment for investigating the pathogenesis of LN and development of effective treatments to a large extent. However, the administration of exogenous IFN-alpha can shorten the onset time of clinical manifestations in lupus-prone mice, thereby enabling the reliable control of LN development. Therefore, this acceleration of LN by IFN-alpha provides a useful model for studying the pathogenesis of LN and evaluating new treatments for this disease (Davidson and Liu 2013).

The deposition of immune complexes in renal tissue activates immune cells, including plasmacytoid dendritic cells, via STAT signaling, which attenuates the downstream expression of genes in the type I IFN pathway (TI-IFN-P) (Banchereau and Pascual 2006). Recently, several studies have demonstrated that resident nephrocytes are also directly activated by immune complexes to produce inflammatory mediators that promote the accumulation of immune cell types (Lema et al. 2001). Resident renal cells (RRCs), including renal mesangial cells (RMCs), are essential for the pathogenesis of LN (Ka et al. 2007; Krötz 2011). RMCs represent a type of RRC that can release type I IFN and aggravate autoimmune renal injury (Fairhurst et al. 2009). Nevertheless, the regulatory mechanism of type I IFN signals in RMCs remains unclear.

Circular RNAs (circRNAs) are unique noncoding RNAs (ncRNAs) expressed widely in human cells and have attracted great interest in recent years (Meng et al. 2017). As an endogenous ncRNA, circRNA is considered to be a key regulator of various biological processes (Ebbesen et al. 2017). In addition, circRNAs have been shown to be associated with cancer generation and development. Based on a previous study on lung cancer, the epithelial–mesenchymal transition and proliferation were shown to be inhibited by hsa_circ_0007059 via microRNA (miRNA)-378 inhibition (Gao et al. 2019). Overexpressed circ_0007059 can inhibit cell proliferation, repress CyclinD1 expression, and elevate p53 expression in A549 and H1975 cell lines. This is accompanied by apoptosis and the increased expression of active caspase-3 and Bax. In oral squamous cell carcinoma, circ_0007059 is associated with prognosis and affected malignant behavior via AKT/mTOR signaling (Su et al. 2019). Circ_0007059 upregulation can inhibit cell growth, invasion, and accelerate apoptosis in SCC15 and CAL27 cell lines. Furthermore, tumor formation studies in nude mice indicated that circ_0007059 has a tumor suppressive effect in vivo. However, a role for circ_0007059 in RMC behavior during nephritis has not been reported. Therefore, the current study aimed to evaluate the expression and function of circ_0007059 in IFN-induced RMCs and human embryonic kidney 293 (HEK293) cells. This will provide further understanding of the molecular mechanisms and pathogenesis of LN and lead to the development of new treatment strategies for lupus.

## Methods

### Patients

We enrolled 30 patients (female 30; age range 19.6–45.0 years; mean age 37.5 years) with SLE from the Affiliated Hospital of Youjiang Medical University for Nationalities from Dec 2015 to Dec 2018. The study was approved by the Ethics Committee of the Affiliated Hospital of Youjiang Medical University for Nationalities (Approval number: YYFY-LL-2020-14). All patients provided written informed consent. Renal biopsy was performed in 18 patients with continuous SLE and active nephritis; 12 were diagnosed with inactive SLE. Partial nephrectomy was performed for renal biopsy, and the tissue was immersed in 4% formaldehyde at room temperature overnight prior to sectioning for in situ hybridization. The patients were diagnosed in accordance with the diagnostic criteria of SLE established by the American College of Rheumatology (Hochberg 1997). We measured the Systemic Lupus Erythematosus Disease Activity Index (SLEDAI) score in patients’ blood. Patients with a score index of < 4 were classified into the inactive disease group, whereas patients with a score index of ≥ 4 were placed into the active disease group. The renal SLEDAI scores were measured as described previously (Pitashny et al. 2007). The proliferation of mesangial cells is considered the primary pathological change in SLE. For this study, we used ten pairs of kidney tissue samples as controls. For pathological examination, parts of the kidney were surgically removed.

### LncRNA microarray

Total RNA from LN samples (n = 7) and the adjacent normal tissue (n = 7) was extracted using TRIzol. We evaluated the RNA quality using Agilent 2100 bioanalyzer (Agilent Technologies), followed by microarray analysis. We used miRNA QC tool software to analyze rRNA (Affymetrix). The rRNA ratio (28S/18S) of all samples in the experiment exceeded 1.8, and the RNA Integrity Number exceeded 8.0. For the LN tissue, we considered a 1.5-fold difference and P < 0.05 as significantly differentially expressed miRNAs.

### Cell culture and stimulation

Human primary RMCs were obtained from ScienCell Research Laboratories, and HEK293 cells were supplied by the American Type Culture Collection (Manassas, VA, USA). The phenotypes of both cell lines were checked daily. Type I IFN (1000 units/ml; PBL Interferon Source) was used for mouse and primary human RMCs.

### Cell transfection

Circ_0007059 (pLCDH-hsa_circ_0007059) and SHP-1 (pcDNA3.1-SHP-1) overexpressing plasmids and the miR-1278 mimic (mimic sense 5′-UAG UAC UGU GCA UAU CAU CUA U-3′) and its related control (NC mimic, 5′-UUG UAC UAC ACA AAA GUA CUG-3′) were obtained from Genscript Co., Ltd. (Beijing, China). Lipofectamine 3000 (Invitrogen, Carlsbad, CA, USA) was used to transfect RMCs and HEK293 cells based on the manufacturer’s instructions. The transfected RMCs and HEK293 cells were collected for subsequent experiments 40 h after transfection.

### Real-time quantitative polymerase chain reaction (RT-qPCR)

The RNeasy Mini Kit (Qiagen, Hilden, Germany) was used to isolate total RNA as per the manufacturerʼs instructions. Quality of total RNA was checked by Nanodrop® ND-1000 UV–Vis Spectrophotometer. RNA was treated with 3 U/mg RNase R (Epicenter, Madison, WI) for 15 min at 37 °C to digest linear RNA. The Prime Script RT Master Mix (Takara Bio Inc., Kusatsu, Japan) with oligo (dT) or random primers was used to reverse-transcribe the treated RNA (500 ng). Quantitative PCR was performed using 2 × PCR Master Mix (Thermo Fisher Scientific, Waltham, MA). The relative expression of the different genes was calculated by the ΔΔCt method using GAPDH and U6 snRNA expression as internal controls for mRNA and miRNA, respectively. The following primers were used for qPCR: circ_0007059 F, 5′-GAG ACA GTA GCC ATC CAG C-3′; hsa_circ_0007059 R, 5′-TGA TCT GAG TCC AGG TGT T-3′; GAPDH F, 5′-TCA AGG CTG AGA ACG GGA AG-3′; GAPDH R, 5′-TCG CCC CAC TTG ATT TTG GA-3′; and U6 F, 5′-CTC GCT TCG GCA GCA CA-3′; U6 R, 5′-AAC GCT TCA CGA ATT TGC GT-3′.

### Cell Counting Kit-8 (CCK-8) assay

Cells were plated into 96-well plates at a density of 2 × 10^3^ cells/well. At 0, 24, 48, and 72 h, CCK-8 (10 µl) (Biyuntian Biotechnology Co. Ltd., Shanghai, China) was added with fresh medium (90 μL) to each well and incubated the plate for 1 h. Optical density (OD) was measured with a microplate reader at 450 nm and 630 nm. The analysis of OD values and graph preparation was performed using GraphPad 5.0 (GraphPad Software, La Jolla, CA).

### MTT assay

The MTT assay was used to assess cell viability. MTT (20 μL of 0.5 mg/mL) was added to the cells after the supernatant was discarded to dissolve the formazan dye. DMSO (150 μL) was added to each well and incubated for 10 min. OD was measured at 540 nm using Infinite M200 microplate reader (Tecan, Männedorf, Switzerland).

### Flow cytometry

Trypsin was used to digest cells after culturing for 48 h. The apoptotic rate was assessed using Annexin V FITC/propidium iodide (PI) Apoptosis Assay Kit (Biyuntian Biotechnology Co. Ltd.) as per the manufacturer’s instructions. The cells were suspended in 1 × Annexin V binding buffer and Annexin V (5 μL) and PI (1 μL) were added to the cell suspension (100 μL) and mixed. The mixture was incubated in darkness at room temperature for 15 min, and then 1 × Annexin V binding buffer (400 μL) was added the each sample to stop the reaction. The apoptosis rate was measured using FACSCalibur flow cytometer (BD Biosciences, Franklin Lakes, NJ).

### Western blot (WB) analysis

Extracts of cells and specimens were prepared in RIPA buffer (Beyotime Biotechnology) at 4 °C. WB was performed with the cell extracts using commercially available primary antibodies. Primary antibodies used in this study includes: Antibodies to GAPDH (1:5000, ab8245, Abcam), Caspase-3 (1:1000, ab4051, Abcam), Caspase-9 (1:1000, ab25758, Abcam), cleaved Caspase-3 (1:1000, ab2302, Abcam), cleaved Caspase-9 (1:1000, ab2324, Abcam), SHP-1 (1:2000, AB227503, Abcam), STAT3 (1:2500, ab31370, Abcam), and phosphor STAT3 (1:500, ab7315, Abcam). A combination of horseradish peroxidase-conjugated goat anti-rabbit (1:1,000; A0208) and goat anti-mouse secondary antibodies (1:1000; A0216, all from Beyotime Biotechnology) were used to detect the immunoreactive bands; development was performed using the Millipore chromogenic chemiluminescence detection kit (Millipore Sigma, Burlington, MA).

### TargetScan prediction

Circ9119 targets were identified by the prediction algorithm StarBase (http://starbase.sysu.edu.cn/), and miR-21 targets were identified by TargetScan (http://www.targetscan.org). We listed the results based on the prediction of targeting efficacy, and they were ranked according to a conservative probability of a target (Friedman et al. 2009).

### Dual luciferase reporter assay (DLRA)

Regulatory interactions among circ_0007059, miR-1278, and SHP-1 were assessed by luciferase reporter assays and included wild-type and mutant 3′-untranslated region (UTR) of circ_0007059, and SHP-1. We used an miR mimic-NC and luminescence vectors to transfect cells, which were analyzed 36 h after incubation.

### Mouse model

LN accelerated in (NZB × NZW)F1 mice with IFNα-expressing adenovirus. C57BL/6, NZB, and NZW mice were purchased from Vital River Laboratory, and a colony of (NZB × NZW)F1 mice was developed in a specific pathogen-free barrier facility. Glomerulonephritis was accelerated in the (NZB × NZW)F1 mouse model by administration of IFNα-expressing adenovirus, as described previously (Liu and Davidson 2013). Briefly, 8–10-week-old (NZB × NZW)F1 mice were treated with a single intravenous (IV) injection of 10^9^ particles of IFNα5 adenovirus (ViGene Biosciences) or a control adenovirus.

(NZB × NZW)F1 mice were injected IV with Lentiviral-circ_0007059, Lentiviral-miR-1278, Lentiviral-SHP-1 (RiboBio) or control agomir in Entranster in vivo Transfection Reagent (Engreen) on 3 consecutive days 3 and 5 weeks after IFNα5 adenovirus injection for the prevention assay or 5 and 7 weeks after IFNα5 adenovirus injection for the treatment assay. Urine was measured 3, 5, 7, and 9 weeks after IFNα5 adenovirus injection. Mice were euthanized, and kidneys were obtained 7 weeks after IFNα5 adenovirus injection in the prevention assay or 9 weeks after IFNα5 adenovirus injection in the treatment assay. All experiments complied with the relevant laws and institutional guidelines, as overseen by the Animal Studies Committee of the Affiliated Hospital of Youjiang Medical University (Approval number: 2017060501).

### Statistical analyses

The mean ± standard error of the mean from three independent trials are shown in figures. SPSS 17.0 software was used for the statistical analyses of data. P values were determined using two-tailed Studentʼs *t* tests, and P < 0.05 was considered statistically significant.

## Results

### Circ_0007059 expression is downregulated in LN specimens and IFN-treated RMCs

**Fig. 1:**
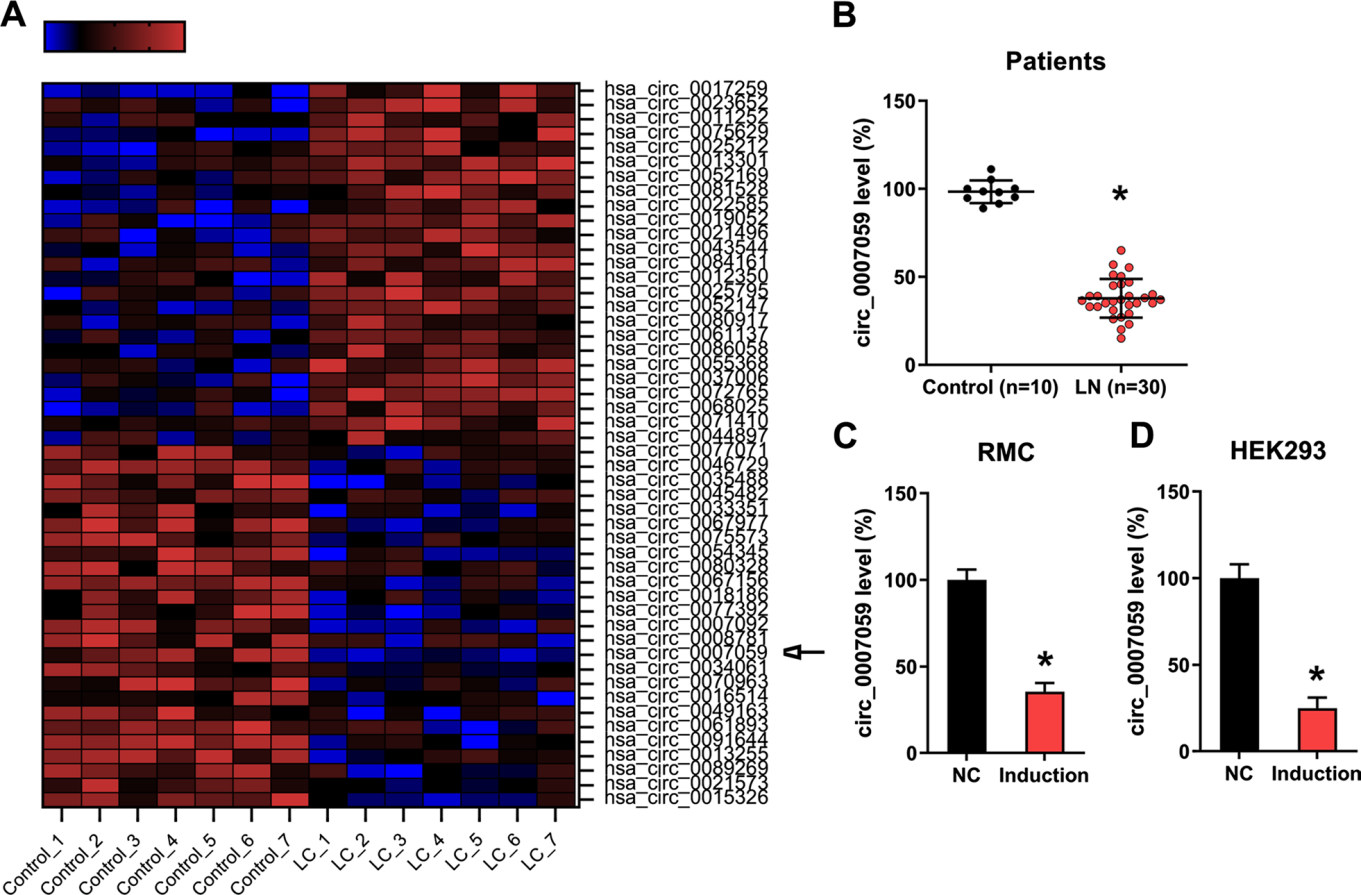
Expression of circ_0007059 in kidneys, IFN-induced RMCs and HEK293 cells, and patients with lupus nephritis (LN). **A** microarray analysis revealed differentially expressed genes between kidney samples from patients with LN (n = 7) and normal healthy tissue (n = 7) (biological replicates, 7; technical replicates, 1; repeat time, 3). **B** the expression of circ_0007059 in renal biopsy samples from patients with LN (n = 30) and surrounding normal tissue samples (n = 10) by RT-qPCR (biological replicates, as indicated; technical replicates, 3; repeat time, 3). **C**, **D** treatment of RMCs and HEK293 cells with IFN (1,000 units/mL) for 24 h. RT-qPCR was used to measure the expression of circ_0007059 (biological replicates, 3; technical replicates, 2; repeat time, 3). Results are showed as the mean ± SEM for biological replicates. *P < 0.05 vs. the indicated group

To study the role of circRNAs in the progression of LN, abnormal circRNA expression was evaluated in renal biopsy samples from patients with SLE using microarray analysis. The expression of several miRNAs in the kidneys of patients with LN was significantly lower than that of the control group, particularly for circ_0007059 (Fig. 1A). To confirm these results, RT-qPCR analysis was performed to measure circ_0007059 expression in samples from patients with SLE (n = 30) and normal samples (n = 10). The results indicated that the level of circ_0007059 in LN samples was lower than that of healthy controls (Fig. 1B). Because type I IFN is essential for LN in humans and mice, we treated RMCs and HEK293 cells with IFN to induce the LN phenotype in these models (Wolf et al. 2018). Of note, IFN treatment reduced the expression of circ_0007059 (Fig. 1C, D). Hence, our results suggest that circ_0007059 is involved in LN progression.

### Circ_0007059 overexpression increases viability and suppresses apoptosis and IFN signaling in RMCs and HEK293 cells

**Fig. 2:**
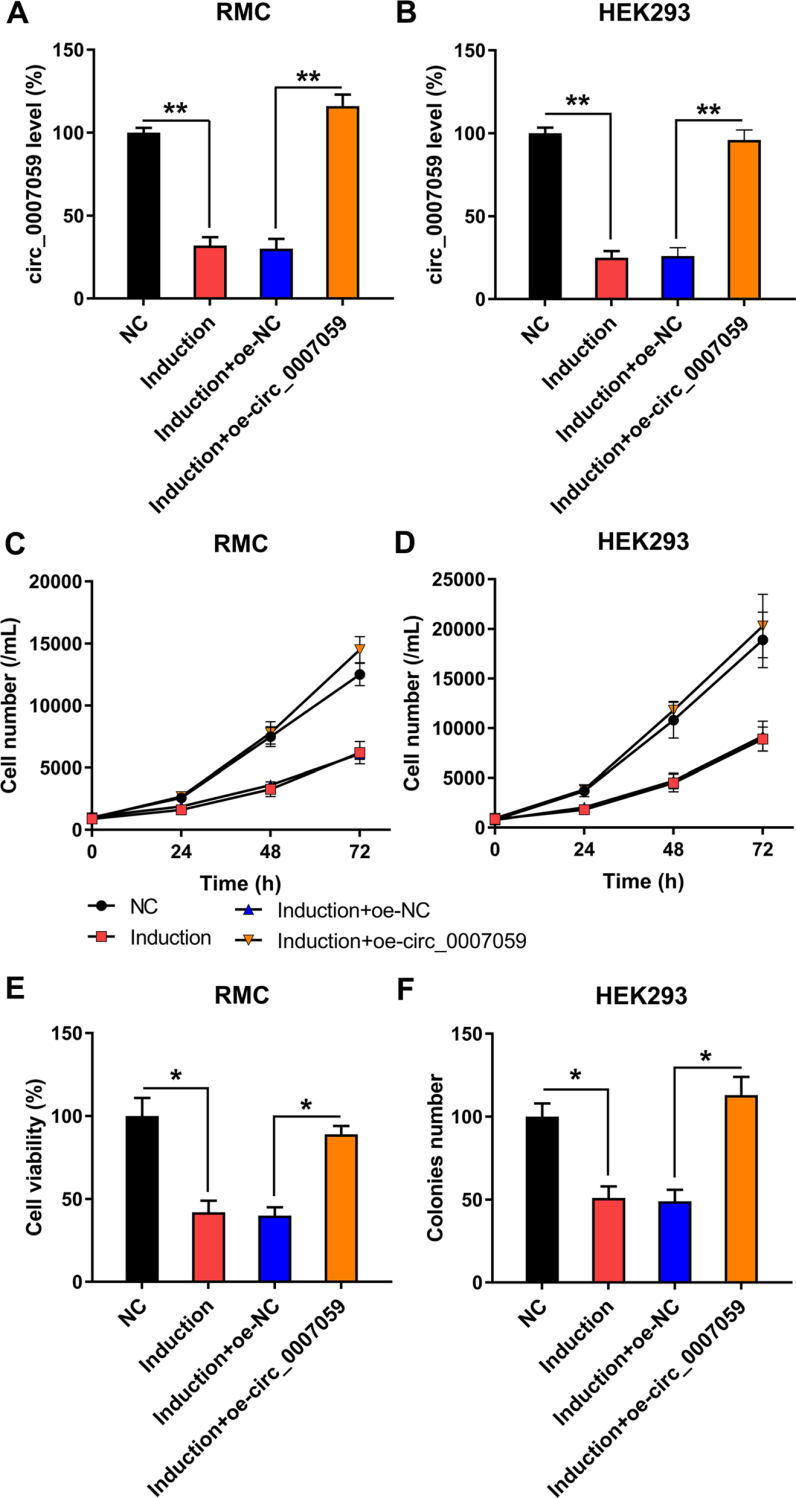
Influence of circ_0007059 overexpression on the cell viability of IFN-induced RMC and HEK293 cells. RMC and HEK293 cells were transfected with a circ_0007059-overexpressing vector or NC vector for 24 h, followed by treatment with 1,000 units/mL IFN for 24 h. **A**, **B** Expression level of circ_0007059 in RMC and HEK293 cells was detected by RT-qPCR (biological replicates, 3; technical replicates, 2; repeat time, 3). **C**, **D** A CCK-8 assay was used to measure the cell proliferation rate at 24, 48, and 72 h after transfection (biological replicates, 1; technical replicates, 3; repeat time 3). **E**, **F** An MTT assay was used to determine cell viability, whereas untreated cells served as controls (biological replicates, 1; technical replicates, 3; repeat time, 3). Results are expressed as the mean ± SEM for technical replicates. *P < 0.05, **P < 0.01 vs. the indicated group

To analyze the effect of circ_0007059 on the viability of IFN-treated RMCs and HEK293 cells, we transfected cells with a circ_0007059-overexpressing vector or control vector. RT-qPCR data confirmed that the transfection of circ_0007059 resulted in a significant increase in circ_0007059 expression levels (Fig. 2A, B). The CCK-8 assay revealed that RMC and HEK293 cell proliferation were noticeably inhibited at 24, 48 and 72 h after IFN induction, but circ_0007059 expression completely restored proliferation (Fig. 2C, D). Furthermore, as determined by the MTT assay, circ_0007059 overexpression led to a noticeable restoration in cell viability, which was suppressed by IFN-treated RMCs and HEK293 cells (Fig. 2E, F). These results suggested a beneficial role of circ_0007059 on the viability of IFN-treated RMCs and HEK293 cells.

**Fig. 3:**
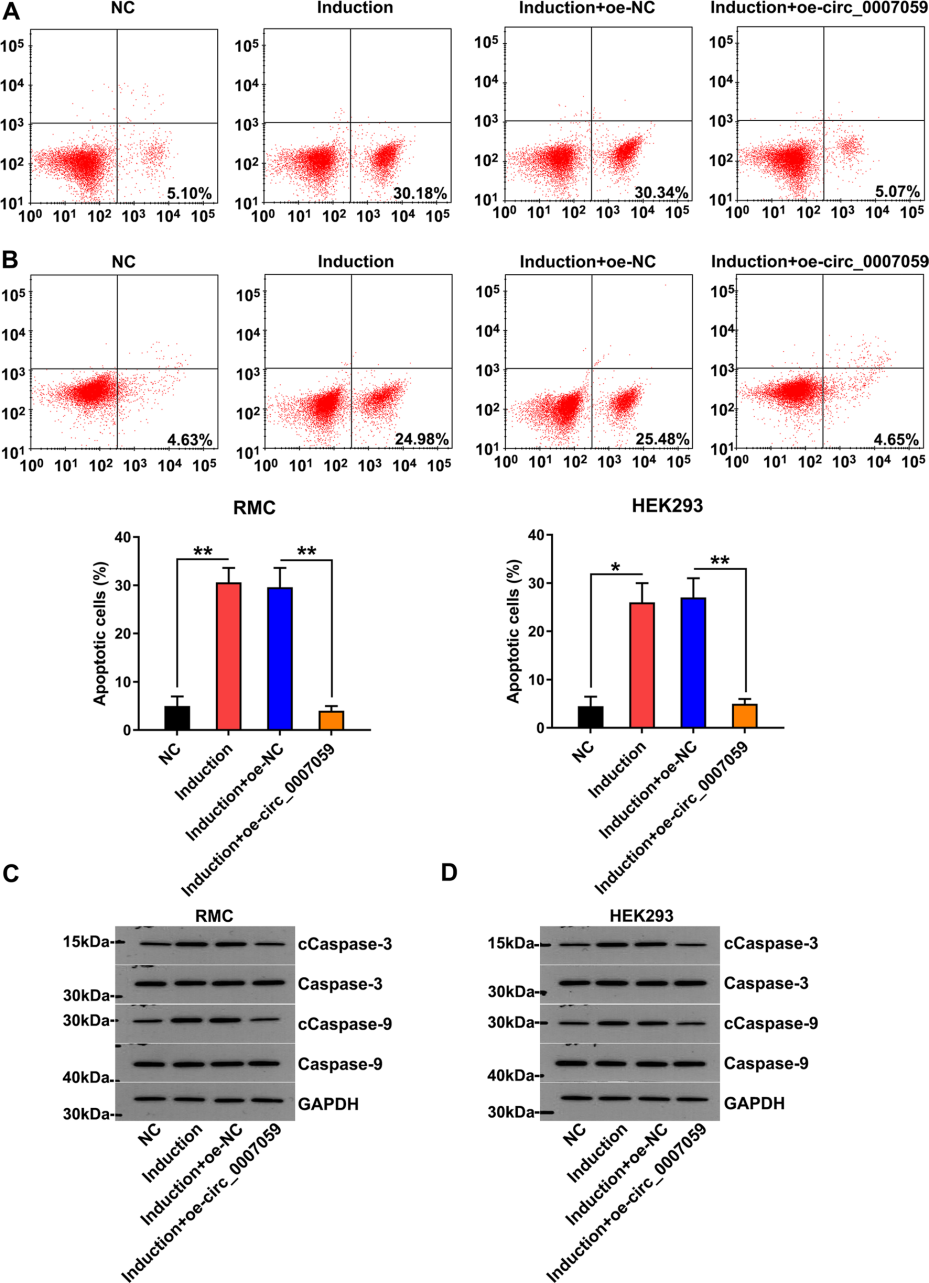
Effect of circ_0007959 overexpression on the apoptosis of IFN-treated RMCs and HEK293 cells. RMC and HEK293 cells were transfected with a circ_0007059-overexpressing vector or NC vector for 24 h, followed by treatment with 1000 units/mL IFN for 24 h. **A**, **B** the number of apoptotic cells was evaluated by flow cytometry. In each plot, the lower right quadrant represents the early apoptotic cells. The analysis of the apoptosis rate in each group is shown in the table below (biological replicates, 2; technical replicates, 3; repeat time, 3). **C**, **D** WB was performed to detect the expression of apoptotic markers, including the cleavage forms of caspase-3 and -9 (biological replicates, 3; technical replicates, 1; repeat time, 3). Results are expressed as the mean ± SEM for technical replicates. *P < 0.05, **P < 0.01 vs. the indicated group

Considering that circ_0007059 restored the IFN-impaired proliferation of RMCs and HEK293 cells, we hypothesized that circ_0007059 may also inhibit cell apoptosis in IFN-induced RMCs and HEK293 cells. First, to test its effects on cell apoptosis, Annexin V/PI flow cytometry indicated significant apoptosis in cells treated with IFN, whereas apoptotic cell proportion was significantly reduced after circ_0007059 overexpression (Fig. 3A, B). In addition, the reduced cleavage of caspase-3 and caspase-9 indicated that circ_0007059 inhibited IFN-induced cell apoptosis (Fig. 3C, D). We also used apoptosis inhibitor QVD to clearly incriminate apoptosis. Caspase inhibitor QVD (100 nM) was administrated in IFN-treated RMC and HEK293 cells, and the expression and cleavage of caspase-3, as well as flow cytometry were performed to determine apoptosis. Expectedly, QVD also ameliorated IFN-caused apoptosis (Additional file 1: Fig. 1A). Our data also showed that QVD treatment contributed to the inhibition of Caspase-3 cleavage (Additional file 1: Fig. 1B), suggesting that circ_0007059 possessed a similar inhibitory effect with QVD on IFN-induced apoptosis.

**Fig. 4:**
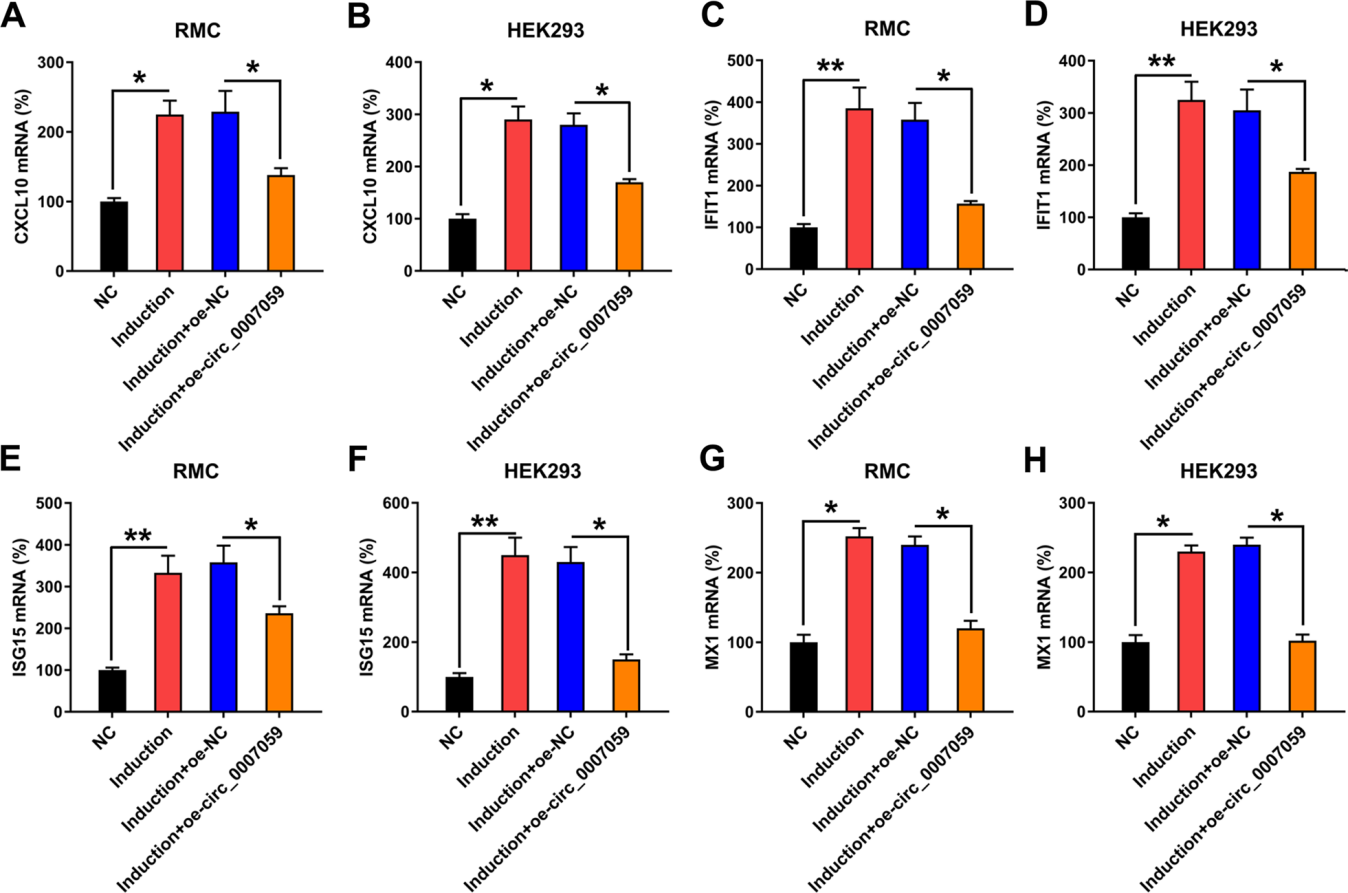
Effect of circ_0007959 overexpression on the apoptosis of IFN-induced genes in RMCs and HEK293 cells. RMC and HEK293 cells were transfected with a circ_0007059-overexpressing vector or NC vector for 24 h, followed by treatment with 1,000 units/mL IFN for 24 h. RT-qPCR was used to determine the expression of mRNA for **A**, **B** CXCL10, **C**, **D** IFIT1, **E**, **F** ISG15, and **G**, **H** MX1 in RMCs and HEK293 cells (biological replicates, 1; technical replicates, 4; repeat time, 3). Values are expressed as the mean ± SEM for technical replicates. *P < 0.05, **P < 0.01 vs. the indicated group

We then tested whether circ_0007059 regulates TI-IFN-P in HEK293 and RMC cells. The expression of several genes induced by IFN was evident following the treatment of RMCs with IFN, including CXCL10, IFIT1, ISG15 and MX1, whereas the overexpression of circ_0007059 consistently decreased the expression of these genes (Fig. 4A, C, E, G). Circ_0007059 exerted the same effect on IFN-treated HEK293 cells (Fig. 4B, D, F, H).

### Circ_0007059 modulates miR-1278 expression, which, in turn, regulates SHP-1 expression

**Fig. 5:**
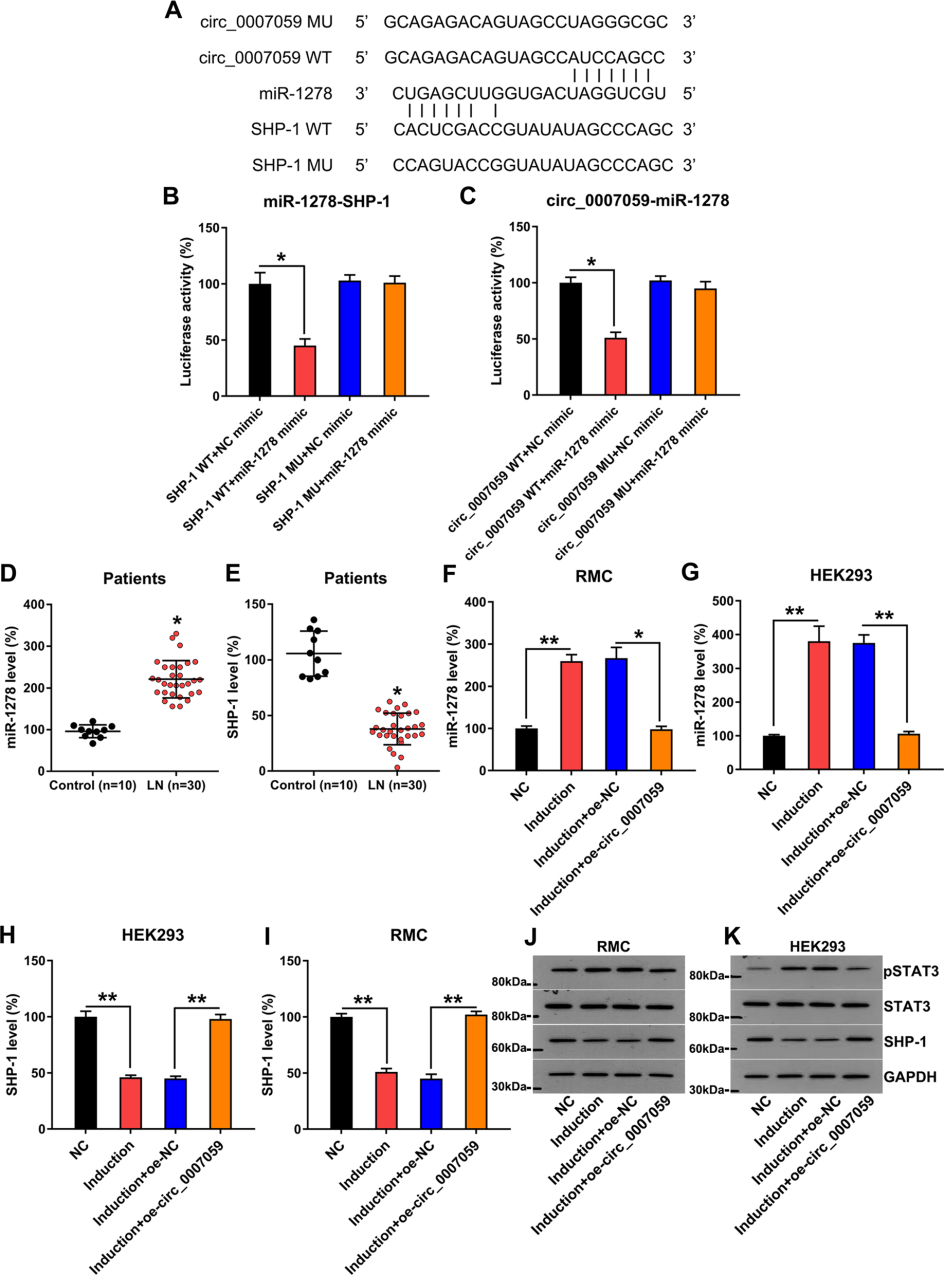
circ_0007059 targeted miR-1278 and miR-1278, in turn, targeted SHP-1. **A** Graphical illustration of the conserved circ_0007059 binding motifs in the 3′-UTR of SHP-1 for miR-1278 and miR-1278-binding motifs. **B**, **C** a luciferase reporter assay was used to measure luciferase activity, including wild-type or mutated (MU) copies of human circ_0007059 and SHP-1 after transfection an miR-1278 mimic in HEK293 cells. Luciferase activity was normalized to that of Renilla luciferase (biological replicates, 1; technical replicates, 3; repeat time, 3). **D**, **E** the expression of miR-1278 and SHP-1 mRNA in renal biopsy samples from patients with LN (n = 30) and adjacent normal tissue samples (n = 10) as measured by RT-qPCR are shown. RMC and HEK293 cells were transfected with a circ_0007059-overexpressing vector or NC vector for 24 h, followed by treatment with 1000 units/mL IFN for 24 h (biological replicates, as indicated; technical replicates, 3; repeat time, 3). **F**, **G** miR-1278 levels in RMCs and HEK293 cells were measured by RT-qPCR (biological replicates, 1; technical replicates, 3; repeat time, 3). **H**, **I** RT-qPCR and **J**, **K** WB was performed to detect SHP-1, STAT3, and phosphorylated STAT3 levels in RMCs and HEK293 cells (biological replicates, 1; technical replicates, 3; repeat time, 3). Results are expressed as the mean ± SEM for technical replicates. *P < 0.05, **P < 0.01 vs. the indicated group

Bioinformatics analysis was used to predict the targets of circ_0007059. We discovered that circ_0007059 targets miR-1278, which, in turn, may target the 3′-UTR of SHP-1, a widely reported mediator of inflammation and apoptosis (Chong and Maiese 2007) (Fig. 5A). Next, we investigated the mechanistic connections between the circ_0007059-miR-1278 and miR-1278-SHP-1 interaction (Fig. 5B, C) using DLRA. When RMCs were transfected with an miR-1278 mimic fused to wild-type circ_0007059 and WT SHP-1, luciferase activity decreased by 55% and 50%, respectively, compared with control cells. We then evaluated the expression of SHP-1 and miR-1278 in samples from patients with SLE (n = 30) and normal samples (n = 10). It was found that the level of miR-1278 was elevated and that of SHP-1 mRNA was reduced in LN samples compared with healthy controls (n = 10) (Fig. 5D, E). The IFN treatment of RMCs and HEK293 cells induced miR-1278 expression, and miR-1278 expression was concomitantly downregulated after circ_0007059 overexpression, confirming that circ_0007059 targets miR-1278 (Fig. 5F, G). We also examined SHP-1 protein and mRNA expression in the IFN-treated RMCs and HEK293 cells to explore the association between SHP-1 and circ_0007059. IFN treatment downregulated SHP-1 expression in RMCs and HEK293 cells, and overexpressed circ_0007059 resulted in increased SHP-1 at both the mRNA and protein levels (Fig. 5H–K). In addition, WB data indicated that STAT3 phosphorylation was significantly reduced by circ_0007059 3 h after transfection (Fig. 5H–K). Thus, our findings suggest that circ_0007059 downregulates miR-1278 expression, resulting in increased SHP-1 expression in IFN-treated cells.

### MiR-1278 upregulation counteracts the impact of circ_0007059 on the viability, apoptosis, and IFN signaling pathway of RMCs and HEK293 cells

**Fig. 6:**
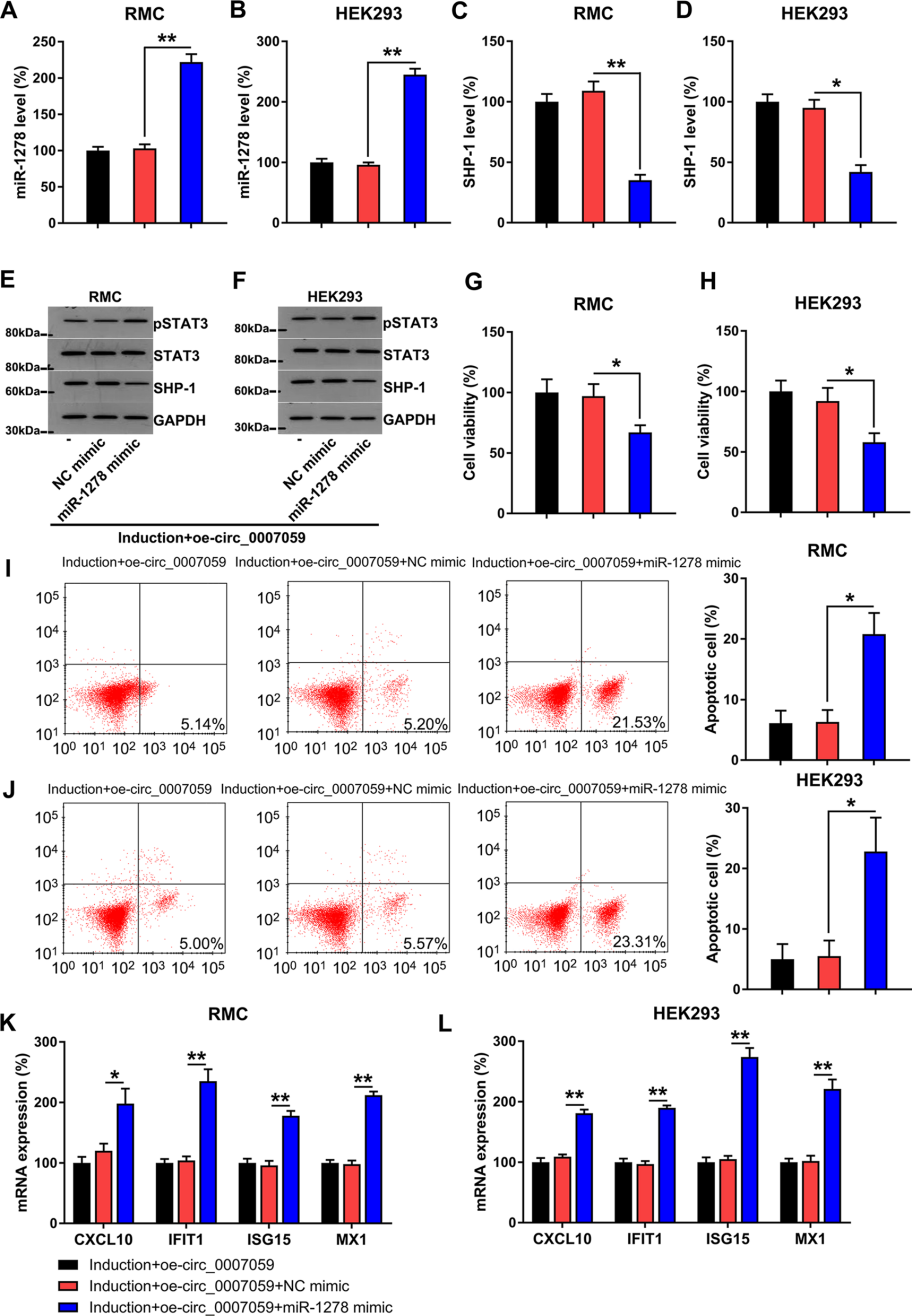
Effect of an miR-1279 mimic on circ_0007059-induced cell viability, apoptosis, and IFN-associated inflammation of RMCs and HEK293 cells. RMC and HEK293 cells were cotransfected with a circ_0007059-overexpressing vector and/or NC/miR-1278 mimic for 24 h, followed by treatment with 1000 units/mL IFN for 24 h. Expression of **A**, **B** miR-1278 or **C**, **D** SHP-1 was evaluated by RT-qPCR in cells with different transfected constructs (biological replicates, 1; technical replicates, 3; repeat time, 3). **E**, **F** WB was performed to detect SHP-1, STAT3, and phosphorylated STAT3 levels in RMCs and HEK293 cells (biological replicates, 1; technical replicates, 3; repeat time, 3). **G**, **H** cell viability of RMCs and HEK293 cells was examined using the MTT assay (biological replicates, 1; technical replicates, 3; repeat time, 3). **I**, **J** the number of apoptotic cells was assessed by flow cytometry (biological replicates, 1; technical replicates, 3; repeat time, 3). **K**, **L** expression of CXCL10, IFIT1, ISG15, and MX1 mRNA in RMCs and HEK293 cells was determined by RT-qPCR (biological replicates, 1; technical replicates, 3; repeat time, 3). Results are expressed as the mean ± SEM for technical replicates. *P < 0.05, **P < 0.01 vs. the indicated group

We evaluated the effect of miR-1278 on circ_0007059-regulated viability, apoptosis, and IFN-related signaling in IFN-treated RMCs and HEK293 cells. The miR-1278 mimic was cotransfected into RMCs and HEK293 cells with a circ_0007059-overexpressing vector to test the effects of miR-1278 upregulation. We confirmed that miR-1278 levels were upregulated with circ_0007059 overexpression in these INF-treated cells (Fig. 6A, B). miR-1278 upregulation also reduced SHP-1 mRNA and protein expression and increased STAT3 phosphorylation (Fig. 6C–F). The MTT assay indicated that transfection with an miR-1278 mimic led to a reduction of viability in IFN-treated RMCs and HEK293 cells (Fig. 6G, H). In addition, Annexin V/PI flow cytometry indicated that miR-1278 upregulation reduced the number of apoptotic RMCs and HEK293 cells (Fig. 6I, J). With respect to IFN-induced signaling, CXCL10, IFIT1, ISG15, and MX1 expression was significantly restored by miR-1278 in RMCs and HEK293 cells with overexpressed circ_0007059 (Fig. 6K, L). These results suggested that circ_0007059 plays a regulatory role by attenuating miR-1278 expression.

### Overexpressed SHP-1 decreases apoptosis and inflammation and promotes proliferation in IFN-treated RMCs and HEK293 cells

**Fig. 7:**
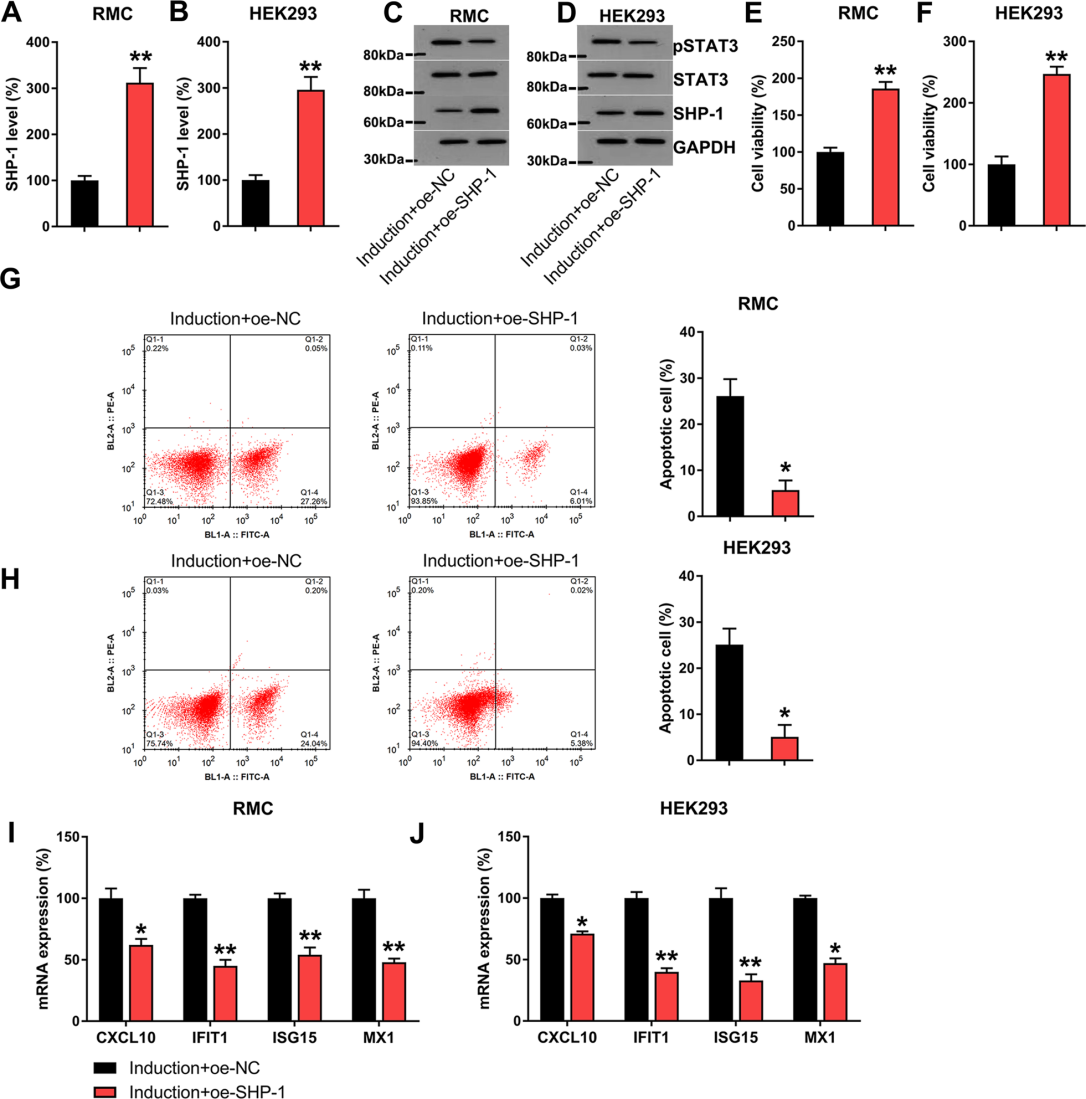
Influence of SHP-1 overexpression on viability, apoptosis, and IFN-associated inflammation of RMCs and HEK293 cells. RMC and HEK293 cells were transfected with an SHP-1-overexpressing vector or NC vector for 24 h, followed by treatment with 1,000 units/mL IFN for 24 h. Expression of (**A**, **B**) SHP-1 was measured in cells with different transfection constructs by RT-qPCR (biological replicates, 2; technical replicates, 3; repeat time, 3). **C**, **D** WB was performed to detect SHP-1, STAT3, and phosphorylated STAT3 levels in RMCs and HEK293 cells (biological replicates, 1; technical replicates, 3; repeat time, 3). **E**, **F** cell viability of RMCs and HEK293 cells were determined by MTT assay (biological replicates, 1; technical replicates, 3; repeat time, 3). **G**, **H** the number of apoptotic cells was evaluated by flow cytometry (biological replicates, 1; technical replicates, 3; repeat time, 3). **I**, **J** expression of mRNA for CXCL10, IFIT1, ISG15, and MX1 gene in RMCs and HEK293 cells as determined by RT-qPCR (biological replicates, 2; technical replicates, 3; repeat time, 3). Results are expressed as the mean ± SEM for technical replicates. *P < 0.05, **P < 0.01 vs. the indicated group

To study the effect of SHP-1 on IFN-treated RMCs and HEK293 cells, STAT3 protein was overexpressed in IFN-treated cells. At first, elevated SHP-1 expression was observed in the SHP-1 overexpression groups (Fig. 7A–D), whereas STAT3 phosphorylation was downregulated in the SHP-1 overexpression groups (Fig. 7C, D). To further evaluate the effect of SHP-1 on cell viability and apoptosis in IFN-treated cells, we assessed these processes by MTT assay and Annexin V/PI flow cytometry. SHP-1 overexpression resulted in a noticeable increase in cell viability and caused a reduction in apoptotic cell proportion compared with the NC group (Fig. 7E–H). Moreover, SHP-1 overexpression resulted in the downregulation of CXCL10, IFIT1, ISG15, and MX1 expression in IFN-treated RMCs and HEK293 cells (Fig. 7I, J).

### Circ_0007059 prevents LN development through mediating miR-1278 and SHP-1

**Fig. 8:**
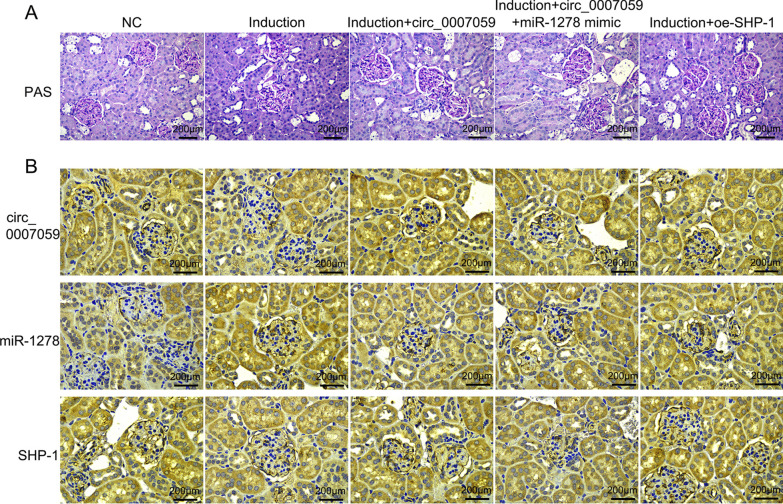
Efficacy of circ_0007059 overexpression in treating IFNα-accelerated LN. **A** PAS-stained kidney sections were analyzed for renal lesion scores, which showed that circ_0007059 agomir ameliorated IFNα-accelerated LN in mice. **B** Histologic analysis of circ_0007059, miR-1278, and SHP-1 protein expression in mice. (Biological replicates = 2; technical replicates = 2, repeat time = 2)

To investigate the potential capacity of circ_0007059, miR-1278, and SHP-1 in preventing or treating LN, Lentiviral-circ_0007059, miR-1278 mimic and SHP-1 were administered to IFNα5 adenovirus-treated (NZB × NZW)F1 mice via tail vein injection. In this study, histologic examination (PAS staining) revealed that IFNα5 adenovirus-treated (NZB × NZW)F1 mice developed severe glomerulonephritis. Notably, Lentiviral-circ_0007059 administrated mice had healthy glomeruli and no significant mesangial hypercellularity or glomerular enlargement. However, upregulation of miR-1278 promoted the glomeruli lesion in the renal. In contrast, overexpression of SHP-1 further ameliorated the severe glomerulonephritis caused by IFNα5 (Fig. 8A). IHC staining showed that renal circ_0007059 was significantly decreased in the mice with IFNα5 induction, while Lentiviral-circ_0007059 administration obviously upregulated the level of circ_0007059 and SHP-1, and downregulated miR-1278 in kidney. Furthermore, treatment of Lentiviral-miR-1278 promoted expression of miR-1278 and reduced level of SHP-1. In addition, Lentiviral-SHP-1 induced an upregulation of SHP-1 in the kidney of IFNα5 adenovirus-treated (NZB × NZW)F1 mice  (Fig. 8B). These data were consistent with in vitro experiment.

## Discussion

Abnormal miRNA expression is related to liver disease severity, for example, in SLE combined with active LN kidneys (Lorenzen et al. 2011; Dai et al. 2009). Therefore, we used miRNA arrays to analyze differential circRNA expression in LN livers and renal biopsy specimens. The expression of circ_0007059 in the renal tissue of patients with LN was significantly lower than that of control patients. Moreover, the levels of circ_0007059 were reduced in IFN-treated RMCs and HEK293 cells. Circ_0007059, a circRNA discovered recently, is associated with lung cancer (Gao et al. 2019), oral squamous cell carcinoma (Su et al. 2019), and other diseases. In the present study, we found that the overexpression of circ_0007059 ameliorated the detrimental effects of IFN in RMCs and HEK293 cells by targeting the miR-1278-SHP-1-STAT3 signaling pathway.

Regarding the pathogenesis of LN, type I IFN is considered a crucial factor. In addition, the expression of IFN-inducible genes (CXCL10, IFIT1, ISG15, and MX1) is increased in patients with SLE, and their expression levels are associated with the activity and severity of LN. Exogenous IFN-alpha can expedite the development and severity of lupus-prone mice or RMCs, and this discovery implicates type I IFN in the development of LN (Han et al. 2016; Ding et al. 2021). In the present study, IFN-alpha was used to treat RMCs and HEK293 cells and to establish an LN cell model. The impaired cell viability and induced IFN-associated gene expression (CXCL10, IFIT1, ISG15, and MX1) indicate that we successfully established the LN cell model.

To the best of our knowledge, this is the first study to demonstrate a novel regulatory loop of circ_0007059-miR-1278-SHP-1-STAT3 in LN development. To date, studies regarding the function of miR-1278 are scant. A previous study indicated that miR-1278 may be differentially expressed in patients with estrogen receptor-positive breast cancer, but its function in breast cancer was not reported. (Sevinc et al. 2015). In this study, compared with control samples, miR-1278 was elevated in the kidneys of the disease group. The ectopic upregulation of miR-1278 impaired cell viability, induced cell apoptosis, and promoted IFN-associated inflammation in IFN-induced RMCs and HEK293 cells with overexpressed circ_0007059. These results suggest that miR-1278 is involved in cell injury due to IFN treatment and in circ_0007059-mediated cell properties and also filled in a gap in our knowledge of the miR-1278’s function during inflammation and apoptosis.

Another novel observation in this study was that miR-1278 targets the 3′-UTR of SHP-1, whereas the latter deactivated STAT3 through dephosphorylation. As nonreceptor PTPs, SHP-1 (also named PTPN6) and SHP-2 (also named PTPN11) are the critical regulators of cellular development, hyperplasia, apoptosis, metastasis, differentiation, inflammation, and intermediate metabolism (Chong and Maiese 2007). SHP-1 is primarily expressed in hematopoietic cells and epithelial cells and is considered to have a negative regulatory effect on the inflammatory response (Fish et al. 2008). Previous studies have suggested that multikinase inhibitors, including sorafenib (Tai et al. 2014), dovitinib (Chen et al. 2012a), and the Mcl-1 inhibitor SC-2001 (Chen et al. 2012b), regulate the phosphatase activity of SHP-1 to increase their antitumor effects. It was also reported that the overexpression of SHP-1 can eliminate the phosphorylation of STAT3^Tyr705^ by TGFB1 to induce the epithelial–mesenchymal transition, invasion, and migration of HCC cells (Wen et al. 2018). STAT3 is a carcinogenic transcription factor that plays a key role primarily via the phosphorylation of its tyrosine residues and their subsequent dimerization and translocation to the nucleus (Lim and Cao 2006; Schindler et al. 2007). The STAT3 signal is activated in various human malignant tumors and participates in the progression of many cancer cell types (Lee et al. 2019). A recent study reported that STAT3 phosphorylation is associated with apoptosis and inflammation in cadmium-induced testicular toxicity in rats (Fouad et al. 2019). Both SHP-1 and STAT3 have been widely investigated in inflammation and apoptosis, but few studies have focused on its role in LN. In this study, the level of SHP-1 in the kidneys of patients with LN was lower than that of control patients. The passive overexpression of SHP-1 by circ_0007059 or overexpression by transfection is accompanied by restored cell viability, inhibited inflammation, and increased apoptosis in IFN-treated RMCs and HEK293 cells. Meanwhile, the passive downregulation of SHP-1 using an miR-1278 mimic resulted in impaired cell viability, induced apoptosis, and IFN-associated inflammation. We also evaluated the phosphorylation of STAT3 along with SHP-1. Our WB data confirmed that phosphorylated STAT3 levels are negatively correlated with SHP-1 expression. The data suggest that SHP-1-STAT3 signaling participates in the IFN-induced nephritis cell model and exerts a cytoprotective role.

However, there are several limitations in the present study: First, the utilization of IFNα to establish a LN cell model. A study has indicated that TLR7-regulated LN is not in a type I IFN-dependent manner (Wolf et al. 2018), suggesting that IFNα is not exclusive inductive factor during LN development; Second, IFNα was also used in the animal model. Although many studies have demonstrated a successful modeling by IFNα induction (Han et al. 2016; Adalid-Peralta et al. 2008; Haselmayer et al. 2014), the difference of pathogenicity between LN animal and human LN samples is not fully elucidated. Finally, to further confirm the role of SHP-1 during LN development, SHP-1 knockdown or knockout animal should be utilized and observed to establish a LN model.

## Conclusions

In conclusion, the present study is the first attempt to demonstrate a correlation of the circ_0007059-miR-1278-SHP-1-STAT3 regulatory loop with LN development. Circ_0007059, miR-1278, and SHP-1 were indicated as important factors in the pathogenesis of LN. However, the main limitation to our study is that the experiments were performed primarily in an in vitro cell model in response to IFN induction. For the purpose of determining the role of circ_0007059, further studies in knockdown or knockout animal models are required. Our results and conclusions provide novel insights into the pathogenesis of SLE.

## Supplementary Information

**Additional file 1: Figure 1.** Effect of apoptosis inhibitor QVD on IFNα-induced apoptosis. Caspase inhibitor QVD (100 nM) was administrated in IFNα-induced treated cells, and the expression and cleavage of caspase-3, as well as flow cytometry were performed to determine apoptosis. **A** the number of apoptotic cells was evaluated by flow cytometry. (Biological replicates = 1; technical replicates = 1, repeat time = 3). **B** WB was performed to detect levels of cleaved Caspase-3 and Caspase-3 in RMCs and HEK293 cells. (Biological replicates = 1; technical replicates = 1, repeat time = 2).

## Data Availability

Not applicable.

## Abbreviations

CircRNAs: Circular RNAs
IFN: Interferon
RMCs: Renal mesangial cells
LN: Lupus nephritis
SLE: Systemic lupus erythematosus
TI-IFN-P: Type I IFN pathway
RRCs: Resident renal cells
NcRNAs: Noncoding RNAs
SLEDAI: Systemic Lupus Erythematosus Disease Activity Index
HEK293: Human embryonic kidney 293
RT-qPCR: Real-time quantitative polymerase chain reaction
CCK-8: Cell counting Kit-8
PI: Propidium iodide
WB: Western blot
DLRA: Dual luciferase reporter assay
IV: Intravenous
SEM: Standard error of the mean
