# Supplementary figures and images for: Circular RNA-0007059 protects cell viability and reduces inflammation in a nephritis cell model by inhibiting microRNA-1278/SHP-1/STAT3 signaling
Source: Mol Med. 2021 Sep 17;27:113. doi: 10.1186/s10020-021-00372-6 (PMC8447523; doi:10.1186/s10020-021-00372-6)

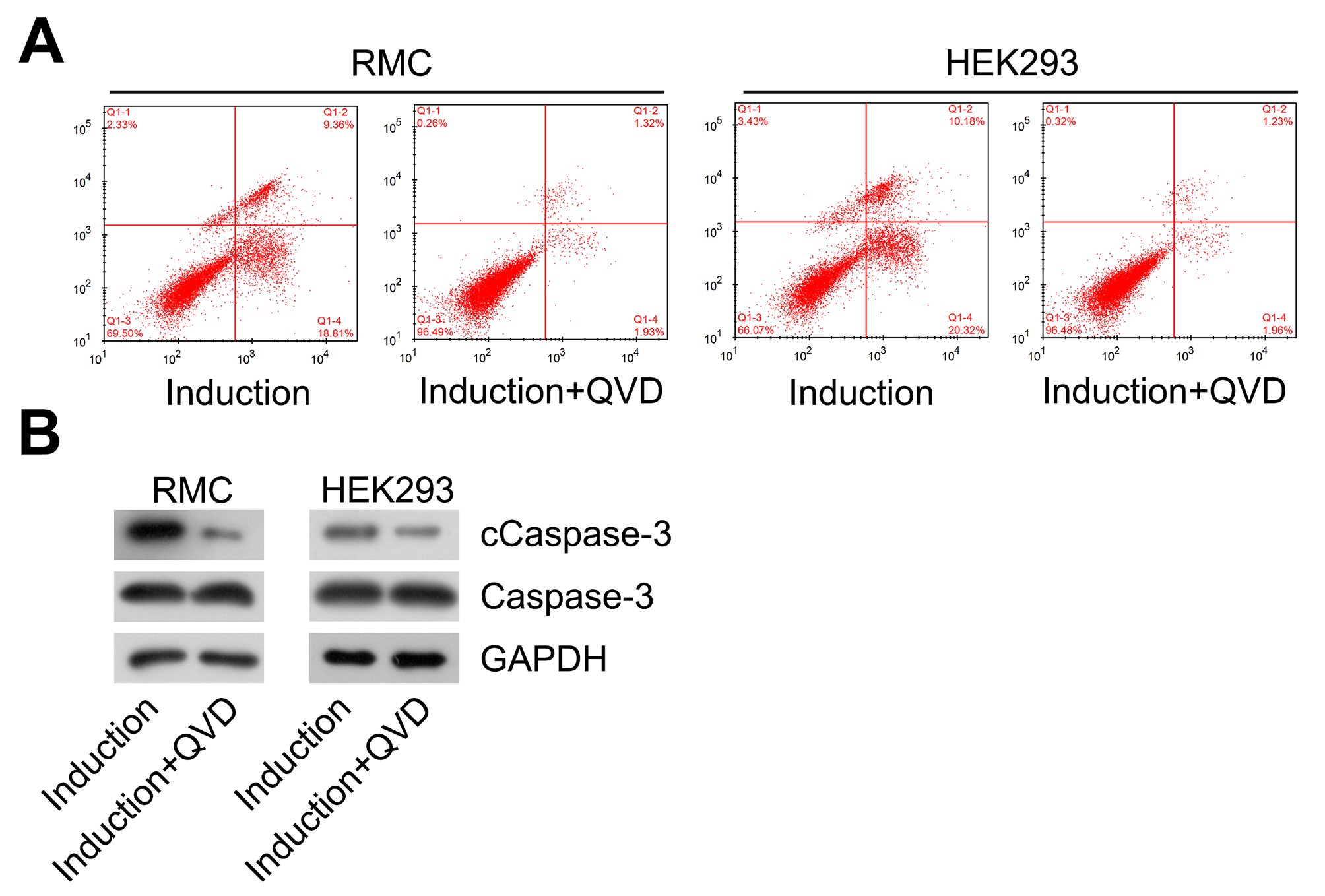

Supplement: Supplementary file 1 — Additional file 1: Figure 1. Effect of apoptosis inhibitor QVD on IFNα-induced apoptosis. Caspase inhibitor QVD (100 nM) was administrated in IFNα-induced treated cells, and the expression and cleavage of caspase-3, as well as flow cytometry were performed to determine apoptosis. A the number of apoptotic cells was evaluated by flow cytometry. (Biological replicates = 1; technical replicates = 1, repeat time = 3). B WB was performed to detect levels of cleaved Caspase-3 and Caspase-3 in RMCs and HEK293 cells. (Biological replicates = 1; technical replicates = 1, repeat time = 2). [file 10020_2021_372_MOESM1_ESM.tif]
